# College and Hospital Notes

**Published:** 1907-02

**Authors:** 


### COLLEGE AND HOSPITAL NOTES.

---

Ground will soon be broken in Buffalo for the erection of the new Marine Hospital which it is expected will be completed in about a year. The hospital will be a three-story and a basement building 114 feet long with an average width of 62 feet, and will be constructed of brick with stone and terra cotta trimmings. A large two-story piazza will be constructed in the rear of the central part of the building. Iron staircases will lead to the upper floors and there will be adequate elevator service.

The basement will contain the steamheating apparatus, the engines for elevators, together with the workshops and essential storerooms. On the right and left of the main entrance will be the hospital offices. The dispensary, laboratory, reception rooms, surgeons' room, bedrooms, and several private rooms for patients will be located on the first floor.

The second floor will contain the wards, dining room, smoking room, officers' wards, and baths, while the third floor will have the surgical ward, sterilising rooms, operating room, etherising room, kitchen, baths, and a few private rooms.

A feature is the location of the kitchen on the third floor. The dormitories of the attendants, located in the upper stories, will contain reading rooms, lounging rooms and separate toilet facilities. The ambulance service will be under contract.

---

The annual report of the New York Post-Graduate Hospital for the year ending October 1, 1906, lately made public, shows a marked increase in the facilities of the institution. The laboratory has been enlarged and an obstetrical ward has been added. For the training school for nurses the building next to the Fahnestock Home has been leased and thoroughly fitted. The nursing staff of the hospital now numbers ninety-nine members. Two wards for diseases of the eye, ear, nose and throat have been equipped.

The patients treated during the year increased to 3,536; dispensary patients, 1,334; visits to the dispensary increased by 6,667. The average daily number of patients in the hospital was 161. The receipts from all sources were $104, 512.04, disbursements $145,549.25, leaving a deficit to be made up by the Post-Graduate Medical School of $41,037.21.

---

The Lackawanna Steel Company has completed the new dispensary on the grounds of the plant at West Seneca (Buffalo). It is a two story brick building completely equipped for operative work. Heretofore, injured workmen were given first aid at the dispensary and when operation was needed it became necessary to remove them to the Moses Taylor Hospital. Now, however, emergency surgery can be done at the new dispensary and the patient later removed to the hospital.

---

Mrs. Charles W. Pardee, president of the Board of Managers of the Buffalo Children's Hospital, has made a gift of a new building to the hospital, assuming the entire expense of structure. The work will be begun at once. The land on which the new hospital is to stand was presented last summer by Miss Martha T. Williams, and adjoins the present hospital building on Bryant street.

---

Dr. Randall announces the appointment of Dr. Harry A. Trick, Dr. Charles P. Chapin and Dr. Clark as trustees and physicians in charge of the Riverside Accident Hospital dispensary. Dispensary hours are from 4 to 6 P. M. daily

